# Characterization of Acetamiprid Biodegradation by the Microbial Consortium ACE-3 Enriched From Contaminated Soil

**DOI:** 10.3389/fmicb.2020.01429

**Published:** 2020-07-08

**Authors:** Bin Xu, Rui Xue, Jie Zhou, Xin Wen, Zhoukun Shi, Minjiao Chen, Fengxue Xin, Wenming Zhang, Weiliang Dong, Min Jiang

**Affiliations:** ^1^State Key Laboratory of Materials-Oriented Chemical Engineering, College of Biotechnology and Pharmaceutical Engineering, Nanjing Tech University, Nanjing, China; ^2^Jiangsu National Synergetic Innovation Center for Advanced Materials (SICAM), Nanjing Tech University, Nanjing, China

**Keywords:** acetamiprid, microbial consortium, community structure, species diversity, degradation pathway

## Abstract

Microbial consortia are ubiquitous in nature and exhibit several attractive features such as sophisticated metabolic capabilities and strong environmental robustness. This study aimed to decipher the metabolic and ecological characteristics of synergistic interactions in acetamiprid-degrading consortia, suggesting an optimal scheme for bioremediation of organic pollutants. The microbial consortium ACE-3 with excellent acetamiprid-degrading ability was enriched from the soil of an acetamiprid-contaminated site and characterized using high-throughput sequencing (HTS). Consortium ACE-3 was able to completely degrade 50 mg⋅L^–1^ acetamiprid in 144 h, and was metabolically active at a wide range of pH values (6.0–8.0) and temperatures (20–42°C). Furthermore, plausible metabolic routes of acetamiprid biodegradation by the consortium were proposed based on the identification of intermediate metabolites (Compounds I, II, III and IV). The findings indicated that the consortium ACE-3 has promising potential for the removal and detoxification of pesticides because it produces downstream metabolites (Compounds I and II) that are less toxic to mammals and insects than acetamiprid. Finally, Illumina HTS revealed that β Proteobacteria were the dominant group, accounting for 85.61% of all sequences at the class level. Among the more than 50 genera identified in consortium ACE-3, *Sphingobium*, *Acinetobacter*, *Afipia*, *Stenotrophomonas*, and *Microbacterium* were dominant, respectively accounting for 3.07, 10.01, 24.45, and 49.12% of the total population.

## Introduction

As one of the most important chloropyridinyl neonicotinoids, acetamiprid plays a major role in crop protection, but its use inevitably causes environmental pollution (Carra et al., 2016; Sun et al., 2017). In fact, acetamiprid residues have often been detected in natural bodies of ground-, surface- and drinking water due to its excessive use to enhance crop production (Cruz-Alcalde et al., 2017). Moreover, although acetamiprid pesticide has been classified as an “unlikely” human carcinogen by the US EPA, it still possesses generalized, non-specific toxicity to mammals. Moreover, acetamiprid residues in soil or on plants have a marked negative impact on non-target species, including livestock, honeybees and humans (Chakroun et al., 2016). It is therefore urgently needed to find an efficient route for the biodegradation of acetamiprid residues, reducing possible ecological risks to the natural environment.

In view of sustainable development goals, indigenous microorganisms are usually used to degrade various environmental pollutants without producing secondary pollution. Accordingly, microbes from different genera were isolated and identified as acetamiprid-biodegraders (Watanabe, 2001; Ju and Zhang, 2014). Examples include *Stenotrophomonas maltophilia* CGMCC 1.1788 (Chen et al., 2008), *Rhodotorula* sp. IM-2 (Dai et al., 2010), *Pigmentiphaga* sp. strain D-2, (Yang et al., 2013), *Pigmentiphaga* sp. strain AAP-1 (Wang et al., 2013a), *Pseudoxanthomonas* sp. AAP-7 (Wang et al., 2013c), *Ochrobactrum* sp. D-12 (Wang et al., 2013b), *Rhodococcus* sp. BCH2 (Phugare and Jadhav, 2015), and *Fusarium* sp. CS-3 (Shi et al., 2018). Among these degraders, strains of *Stenotrophomonas* sp. and *Pigmentiphaga* sp. were respectively able to degrade 58.9% of 570 mg⋅L^–1^ acetamiprid in 8 days and completely degrade 50 mg⋅L^–1^ acetamiprid after 72 h, which was more rapid than other pure bacterial cultures, enabling more efficient bioremediation. However, pure acetamiprid-biodegrading cultures are often unable to perform *in situ* or *on-site* bioremediation with practical value. One of the most important reasons is that pure bacterial cultures cannot effectively mineralize these pesticides due to the formation of derived toxic or inhibitory intermediate metabolites (Copley, 2009). Furthermore, many environmental factors still affect microbial activity (e.g., water, pH, redox potential), leading to possible failure of operation (Liu et al., 2015; Dangi et al., 2019). Therefore, it is urgent to develop reliable bioaugmentation methods for achieving bioremediation of acetamidine in the open environment.

In the open environment, 99% of microorganisms exist in the form of microbial consortia (Ding et al., 2016; Singh and Kashmir, 2016). Compared with pure cultures, microbial consortia possess more flexible and adaptive capabilities to face complex environmental stresses due to combined interactions of diverse species, performing complicated enzymatic catalysis (e.g., intestinal food digestion, wastewater purification, or lignocellulose degradation) (Festa et al., 2013; Dvořák et al., 2017; Mnif et al., 2017). According to Fida et al. (2017) and Su et al. (2019), microbial cells that exhibit low metabolic activity for long-term survival in the viable but non-culturable (VBNC) state still play a crucial role in rapid *in situ* biodegradation of acetamiprid in soil. Therefore, the most appropriate strategy for allowing the emergence of ecologically stable consortia for biodegradation is the development of acclimatized mixed cultures. More recently, increasing numbers of studies are exploring the role of microbial consortia and unculturable microbes in the biodegradation of pollutants. Perruchon et al. (2017b) enriched a bacterial consortium to evaluate their biodegradation capacity for neonicotinoid insecticides under various conditions (pH values, temperatures, and different pollutant concentrations), which indicated that the consortium was more suitable for biodegradation of pollutants in an unstable environment. Similarly, Sharma S. et al. (2014) explored the biodegradation of acetamiprid by another consortium from two soil samples, which achieved excellent degradation performance and realized complete mineralization without producing complex secondary pollutants. This result also indicated that the synergistic interactions between different bacterial strains enabled them to completely mineralize the pollutant.

Here, the microbial consortium ACE-3 was serially enriched from an acetamiprid-contaminated soil. It was able to utilize acetamiprid as the sole carbon source for microbial growth. In addition, the metabolic route of acetamiprid biodegradation by consortium ACE-3 was proposed based on the identification of intermediate metabolites, and the community structure of consortium ACE-3 was determined using high-throughput sequencing (HTS) technology. This study uncovered why and how community structure of mixed consortia affects their biodegradation performance, suggesting an ecologically stable strategy for optimal acetamiprid biodegradation.

Based on these results, we have tested the hypothesis that the community structure of mixed consortia affects their biodegradation performance which suggests an ecologically stable strategy for optimal acetamiprid biodegradation. This was achieved by serial enrichment of the microbial consortium ACE-3 from an acetamiprid-contaminated soil. The consortium was able to utilize acetamiprid as the sole carbon source for microbial growth. In addition, the metabolic route of acetamiprid biodegradation by consortium ACE-3 was proposed based on the identification of intermediate metabolites, and the community structure of consortium ACE-3 was determined using high-throughput sequencing (HTS) technology.

## Materials and Methods

### Chemicals and Media

Chromatography-grade methanol and acetonitrile were purchased from Sigma Aldrich (United States). All molecular biology reagents were purchased from TaKaRa Co., Ltd. (Dalian, China). All other chemicals and reagents were commercially available and were of analytical grade, unless stated otherwise. Acetamiprid (>99% purity) was provided by Jiangsu Academy of Agricultural Sciences, China. The stock solution of acetamiprid (1%, w/v) was prepared in acetone, followed by filtration through a sterile membrane with 0.22 μm pore size. Minimal salt medium (MSM; K_2_HPO_4_ 1.5 g⋅L^–1^, KH_2_PO_4_ 0.5 g⋅L^–1^, NH_4_NO_3_ 1 g⋅L^–1^, MgSO_4_⋅7H_2_O 0.1 g⋅L^–1^, and NaCl 1 g⋅L^–1^, pH7.0) was used to enrich the acetamiprid-degrading culture and for the biodegradation tests.

### Isolation of the Acetamiprid-Degrading Natural Microbial Consortium

Fresh soil (0–15 cm depth) from an acetamiprid-rich environment contaminated for several years was collected at a pesticide factory located in Weifang (Shandong, China). The soil sample was used for enrichment and isolation of the acetamiprid-degrading consortium. A biodegradation system was built in an Erlenmeyer flask (500 mL) containing 5 g soil (dry weight) and 100 mL MSM supplemented with 30 mg⋅L^–1^ acetamiprid as the sole carbon and energy source. The enrichment culture was incubated in a rotary shaker at 180 rpm and 30°C for 6 days. After the degradation efficiency was determined, 10% of the consortium was transferred into fresh MSM containing 50 mg⋅L^–1^ acetamiprid, followed by incubation for another 6 days. The cultivation with increasingly concentrated media was repeated 6 times until the concentration of acetamiprid reached 100 mg⋅L^–1^. The consortium with the highest acetamiprid degradation efficiency was named ACE-3 and used for further study.

### Growth and Biodegradation Assay

When 100 mg⋅L^–1^ acetamiprid was decomposed to a degree of 50%, the cells in the degradation system were collected by centrifugation at 2124 × g and 4°C, washed twice with sterile phosphate buffered saline (PBS, pH 7.0), and re-suspended in sterilized MSM, which was used as inoculum for further study. MSM containing 50 mg⋅L^–1^ acetamiprid was inoculated at a rate of 1%, and un-inoculated MSM was used as the control. Four milliliter samples were drawn at intervals of 24 h to measure the OD_600_, and the remaining acetamiprid concentration in the sample was analyzed by HPLC.

### Effects of Environmental Factors on Acetamiprid Degradation

To optimize the degradation conditions for the ACE-3 consortium, the effects of different factors on acetamiprid degradation were investigated by varying the temperature (20, 25, 30, 37, and 42°C), initial pH (4.0, 5.0, 6.0, 7.0, 8.0, and 10.0), inoculum size (1, 3, 5, and 10%) and initial concentration of acetamiprid (25, 50, 100, 150, and 200 mg⋅L^–1^). All samples were extracted with dichloromethane immediately before HPLC analysis, and the concentration of residual acetamiprid was calculated by comparing the integrated area with that of the reference standard with known concentration. All treatments were performed in triplicate.

### Identification of Metabolites

The consortium ACE-3 was seeded into a 250 mL Erlenmeyer flask containing 100 mL of MSM supplemented with 50 mg⋅L^–1^ of acetamiprid. The samples (acetamiprid degradation percentages of 30, 50, and 70%) were collected by centrifugation at 12,580 × g for 10 min at 4°C. Then, the supernatants were freeze-dried and re-dissolved in 1 mL of chromatography-grade methanol for HPLC-MS analysis. A separation column (internal diameter, 4.6 mm; length, 25 cm) filled with Thermo100-5C18 was used. The mobile phase consisted of 50% water and 50% acetonitrile at a flow rate of 0.6 mL⋅min^–1^. The injection volume was 20 μL, and the column elution was monitored at 247 nm using a UV-900 spectrophotometric detector. The concentration of acetamiprid was calculated by comparing the area under the peak with that of the standard solution (Wang et al., 2013c). The LOD (limit of detection) and LOQ (limit of quantification) of the methods were 1.0 × 10^–7^ mg and 0.002 mg⋅L^–1^, respectively (*R*^2^ = 0.9982). The metabolites were identified by MS (G6410B Triple Quad Mass; Agilent, United States), with electrospray ionization in positive ion mode, and scanned in the normal mass range from 30 m/z (mass to charge ratio) to 300 m/z. Characteristic fragment ions were detected using second-order MS as described before (Zhou et al., 2014).

### Analysis of the Microbial Community by High-Throughput Sequencing

The metagenomic DNA of consortium ACE-3 was extracted using the FastDNA Spin Kit for Soil (MP Biomedicals, United States). The DNA samples were stored at -20°C for subsequent PCR amplification. The universal primers 515F (5′-CCTACGGGAGGCAGCAG-3′) and 907R (5′-TTACCGCGGCTGCTGGC-3′) with different barcodes were used to amplify the V4-V5 region of the 16S rRNA gene (Dong et al., 2017). The 50 μL PCR reaction mixtures consisted of 1 μL DNA template, 25 μL Primer Star Max, 1 μL of each primer (515F and 907R), and 22 μL of double distilled water. The PCR program encompassed an initial denaturation at 94°C for 2 min, followed by 30 cycles of 30 s at 94°C, 30 s at 55°C, and 45 s at 72°C each, and a final extension at 72°C for 10 min. The PCR products were recovered using the AxyPrep DNA gel Recovery Kit (AXYGEN, China), eluted with Tris-HCl, and visualized via 2% agarose gel electrophoresis. The resulting final high-quality sample was used for HTS.

### Sequencing and Analysis

The PCR products were sent to Novogene Co., Ltd. (Beijing, China) for HTS of the 16S rDNA. According to the sequencing requirements of two samples, the Illumina PE250 library was constructed and sequenced. The PE reads obtained by Illumina PE250 sequencing were first spliced according to overlaps, after which the quality of the sequences was controlled and filtered to achieve the requirements of sequence analysis. The raw sequencing data was deposited in the NCBI SRA database under the accession number PRJNA606871.

The UPARSE-OTU algorithm (version 7.1) of the Usearch software platform was used to allocate the operational taxonomic units (OTUs) to High Identity with Tolerance (CD-HIT) groups based on 97% sequence similarity using the cluster database. PCR-generated chimeras were detected and removed using the Uchime method (version 4.2.40; Edgar, 2013). Based on the results of OTU analysis, we calculated the alpha diversity of single samples, including the Chao, Shannon and Simpson indices, using Mothur software (version 1.34.0; Schloss and Westcott, 2011). Classification was conducted using the RDP-II classifier of the ribosome database project (RDP) and the National Center for Biotechnology Information (NCBI) BLAST algorithm at http://www.ncbi.nlm.nih.gov/blast/. The construction of phylogenetic trees was conducted using MEGA software version 7.0 (Kumar et al., 2016). Bootstrap analysis with 1000 replicates was applied to assign confidence levels to the nodes in the phylogenetic trees.

## Results and Discussion

### Degradation of Acetamiprid by the Microbial Consortium ACE-3

**FIGURE 1:**
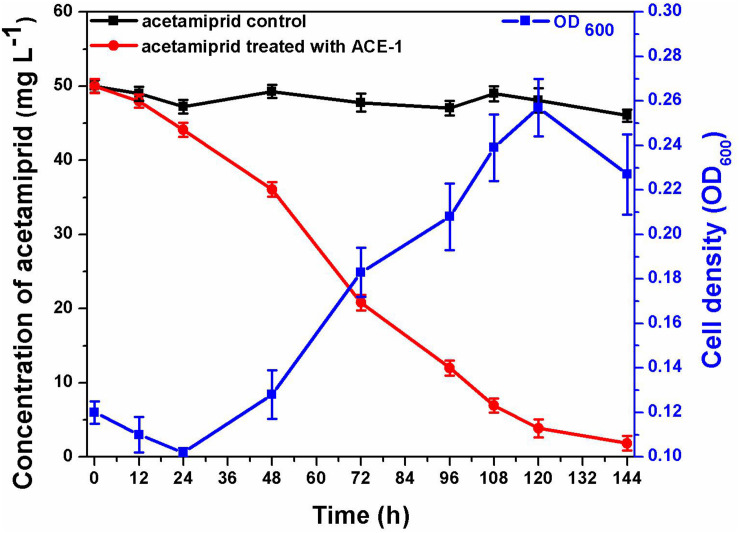
Degradation of acetamiprid by consortium ACE-3 and the corresponding growth curves. The data represent the means ± standard deviations of biological triplicates.

The consortium ACE-3 was successfully enriched by serial selection from acetamiprid-contaminated soil after seven rounds of culture with increasing concentrations of acetamiprid as the sole carbon and energy source. As most bacteria cannot be cultured on conventional bacteriological media because they enter into the VBNC state under harsh environmental conditions (Fida et al., 2017), the growth curve was determined to ensure that the microbial consortium enriched for continuous degradation of acetamiprid was culturable. As shown in Figure 1, consortium ACE-3 was able to degrade 96% of 50 mg⋅L^–1^ acetamiprid within 144 h, while the cell density (OD_600_) increased roughly 3-fold (0.12–0.23, approximately 1.6 × 10^6^–4.8 × 10^6^ cfu⋅mL^–1^). Interestingly, degradation of acetamiprid by consortium ACE-3 was initially slow, with a degradation ratio of 12% within 24 h, possibly due to toxicity of acetamiprid for the microbial population. Subsequently, the rates of acetamiprid degradation and cell growth increased once consortium ACE-3 was well-adapted to the acetamiprid-containing medium. As anticipated, the controls without inoculation with consortium ACE-3 or the addition of acetamiprid resulted in no significant degradation of acetamiprid and nearly negligible bacterial growth, respectively. These results confirmed that consortium ACE-3 possesses the ability to degrade and utilize acetamiprid as the sole carbon source for microbial growth.

Although many acetamiprid-degrading microorganisms have been isolated and studied, cultures of single strains are usually not ecologically stable in practice. Table 1 lists the pure and mixed bacterial cultures used to degrade acetamiprid in earlier studies. In terms of degradation efficiency, both pure and mixed bacteria can achieve efficient biodegradation of acetamiprid. However, from the perspective of the degradation pathway, it is difficult for a single species of bacteria to fully mineralize the secondary metabolites. Therefore, mixed bacterial cultures are more effective for the degradation of environmental pollutants due to synergistic interactions that enable complete mineralization (Chakraborty et al., 2012; Wang et al., 2016; Mori et al., 2017). Application of natural microbial consortia has already shown promise for *in situ* bioremediation of gentamicin, nonyl-phenol, and atrazine-contaminated sites (Baghapour et al., 2013; Bai et al., 2017). This aimed to characterize an acetamiprid-degrading microbial consortium enriched and acclimatized to direct selection pressure for growth on acetamiprid, suggesting a novel operation strategy that can be applied for *in situ* bioremediation of pesticide-contaminated environments.

**TABLE 1:** Bacterial strains capable of degrading acetamiprid.

Type	Microorganisms	Source	Degradation efficiency	Optimal conditions	Mineralization or not	References
Pure	*Ochrobactrum* sp. D-12	Polluted agricultural soil	Degrade acetamiprid with initial concentrations of 3000 mg⋅L^–1^ within 48 h	2–35°C; pH 6–8	No	Wang et al., 2013a
	*Pigmentiphaga* sp. D-2	Wastewater from acetamipridmanufacturing factory	Degrade 0.22 mM acetamiprid to non-detectable level within 72 h	30–42°C; pH 6–7	No	Yang et al., 2013
	*Pseudoxanthomonas* sp. AAP-1	Pesticidecontaminated factory soil	Metabolize 100 mg⋅L^–1^ of acetamiprid within 2.5 h	37°C; resting cells; pH 7	No	Wang et al., 2013b
	*Stenotrophomonas* sp. THZ-XP	Sludge from an acetamipridproducing factory	Degrade 1.0 g.L^–1^ acetamiprid in 25 h	30°C; pH 7	No	Tang et al., 2012
	*Fusarium* sp. CS-3	A pesticide factory	Degrade 99% of acetamiprid with the concentration of 50 mg⋅L^–1^ in 96 h	25–30°C; pH 5–7	No	Shi et al., 2018
Mixed	Unnamed	A local field that had no pesticide application	Degrade 94% of acetamiprid within 15 days	25°C; pH 6	Not mentioned	Liu et al., 2011
	Consortium of Two Soil Isolated *Bacillus* sp.	Sugarcane growing soils	Degrade nearly 90% acetamiprid in clay loam soil under autoclaved and unautoclaved conditions in a time span of 56 days	25 ± 2°C	Yes	Sharma S. et al., 2014
	Phyllosphere bacterial communities	Black land soil	Degrade 11 μg⋅mL^–1^ acetamiprid within 42 days in plant leaves medium	30°C	No	Zhou et al., 2011
	Microbial consortium ACE-3	A pesticide factory	Degrade 96% of the acetamiprid (50 mg⋅L^–1^) within 144 h	25–37°C; pH 5–8	Yes	This work

### Degradation Characteristics

**FIGURE 2:**
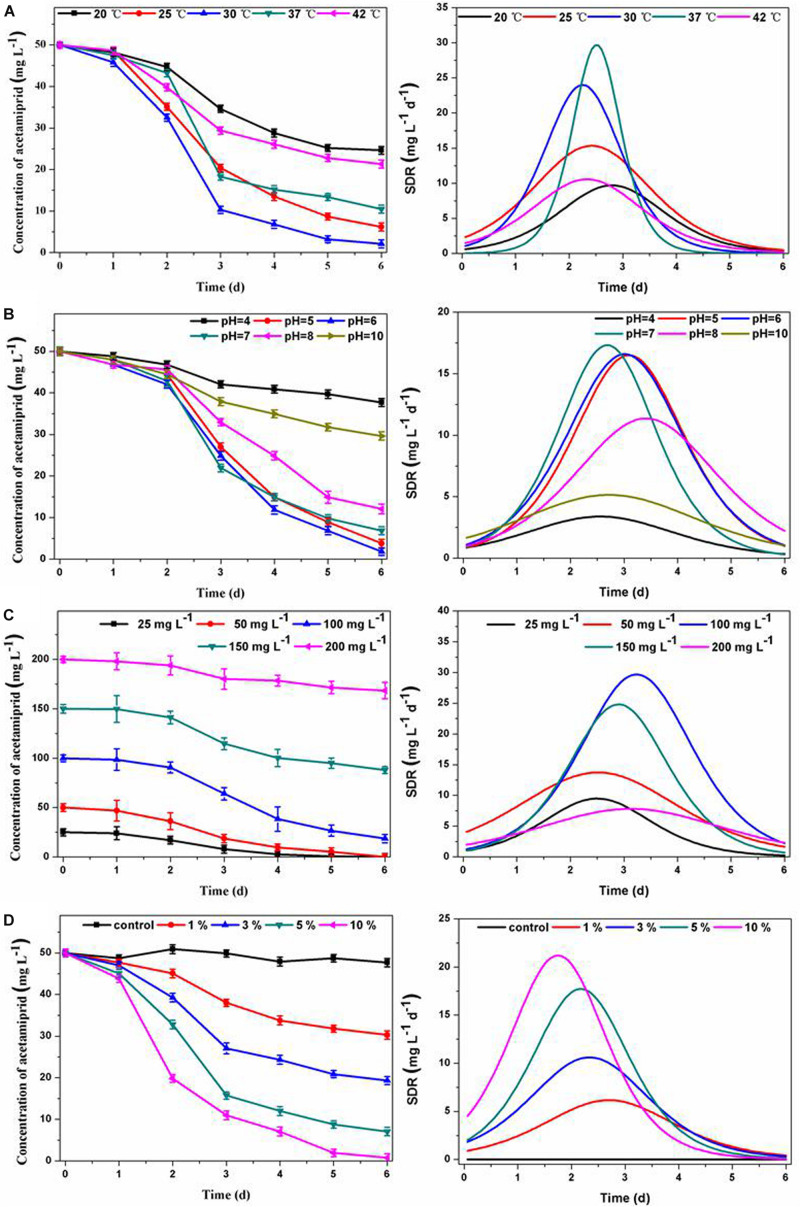
Effects of physicochemical conditions on acetamiprid biodegradation by consortium ACE-3 and degradation kinetics curve of SDR with time-course. **(A)** Temperature; **(B)** pH; **(C)** acetamiprid concentration; **(D)** inoculum size. The data represent the means ± standard deviations of triplicate experiments.

The temperature and pH are significant operation parameters that influence the rate of xenobiotic degradation. As an important parameter, the specific degradation rate (SDR) is often used to evaluate the kinetic process of pollutant degradation over time. The SDR is calculated based on degradation curve fitting, and is used to characterize the amount of acetamiprid degradation per unit of time, representing the degradation rate. As can be seen in Figure 2A, the consortium ACE-3 could degrade acetamiprid at the maximal efficiency at temperatures between 25 and 37°C, while the degradation efficiency was decreased by half at 20 or 42°C. This result indicated that 25–37°C was the optimum temperature range for the consortium to degrade acetamiprid. Similarly, an overly acidic or alkaline environment was not suitable for the biodegradation of acetamiprid by this consortium. The optimum pH for acetamiprid degradation was between 5.0 and 7.0, and the degradation rate decreased drastically at acidic pH values below 4.0 or alkaline pH above 10.0 (Figure 2B). These results indicated that the consortium ACE-3 was ecologically stable, with stable high degradation efficiency and degradation rate over a wide range of environmental changes.

Furthermore, the initial substrate concentration and inoculum size also significantly affected the performance of cell growth and acetamiprid degradation. As shown in Figure 2C, the degradation efficiencies were 99.2 and 97.1% at the initial concentrations of 20 and 50 mg⋅L^–1^, respectively. The degradation efficiency gradually decreased with increasing substrate concentration, most likely due to the toxicity of acetamiprid to bacterial cells. By comparing the maximum degradation rate, it could be seen that when the concentration of acetamiprid reached 200 mg⋅L^–1^, the degradation rate was significantly reduced. The most likely reason for the decrease of the degradation rate is direct inhibition of bacterial growth by the substrate. Similar observations have been reported in the biodegradation of other toxic substances. Correspondingly, when the biomass of the microbial consortium was greater, the acetamiprid degradation rate was significantly increased. As shown in Figure 2D, the acetamiprid degradation ratio at 6 days of incubation increased from 44 to 99.74% when the inoculum size was increased from 1 to 10%. More importantly, as the inoculum size increased, the time to reach the maximum degradation rate was also shorter. These results indicate that the degradation efficiency of acetamiprid is positively associated with the inoculum size of consortium ACE-3.

In summary, the results indicate that consortium ACE-3 could degrade acetamiprid with the highest efficiency under mild conditions. Similarly, the optimal conditions for the degradation of di-n-butyl phthalate (DBP) by consortium LV-1 were pH 6.0 and 30°C (Wang et al., 2017). Moreover, rapid degradation of thiabendazole by a bacterial consortium dominated by the *Sphingomonas* phylotype B13 was observed at slightly acidic pH (5.5–6.5) and moderate temperatures (26–37°C) (Perruchon et al., 2017a). The bacterial consortium DV-AL could degrade 1000 mg⋅L^–1^ of naphthalene within 24 h at an initial pH 8.0 and 37°C (Patel et al., 2012). According to the previous studies listed in Table 1, the optimal conditions of acetamiprid degradation for both pure and mixed cultures were all in the near-neutral range (pH 6 ∼ 8) and mesophilic temperatures (25∼37°C). However, the degradation efficiency changed significantly with the change of temperature and pH. As indicated by Wang et al. (2013b), the degradation process was still possible at pH 5, even if the degradation ratio was significantly reduced from over 80% at pH 7 to around 40%. Also, changes of temperature can have a similar effect. In this study, acetamiprid could be degraded by consortium ACE-3 at a broader range of temperatures and pH values, making such mixed cultures applicable to the bioremediation of various acetamiprid-contaminated environments.

### Identification of Metabolites

**FIGURE 3:**
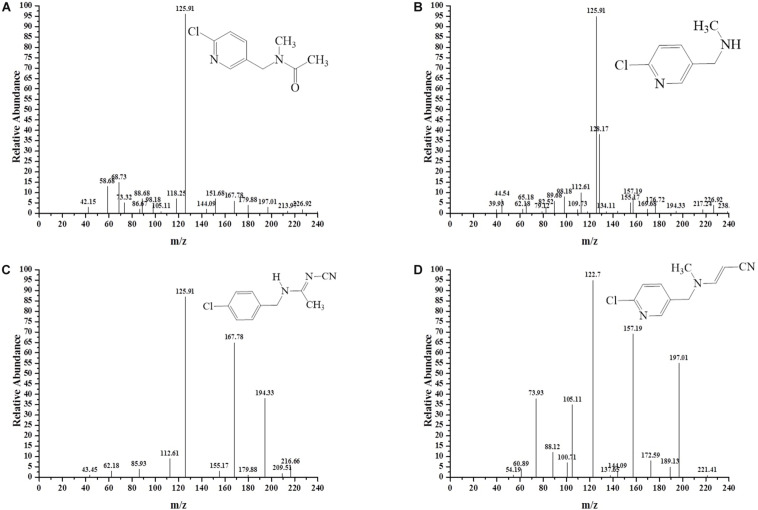
MS/MS identification of the metabolites produced during acetamiprid degradation. Second-order mass spectra of **(A)** compound I; **(B)** compound II; **(C)** compound III; **(D)** compound IV.

**FIGURE 4:**
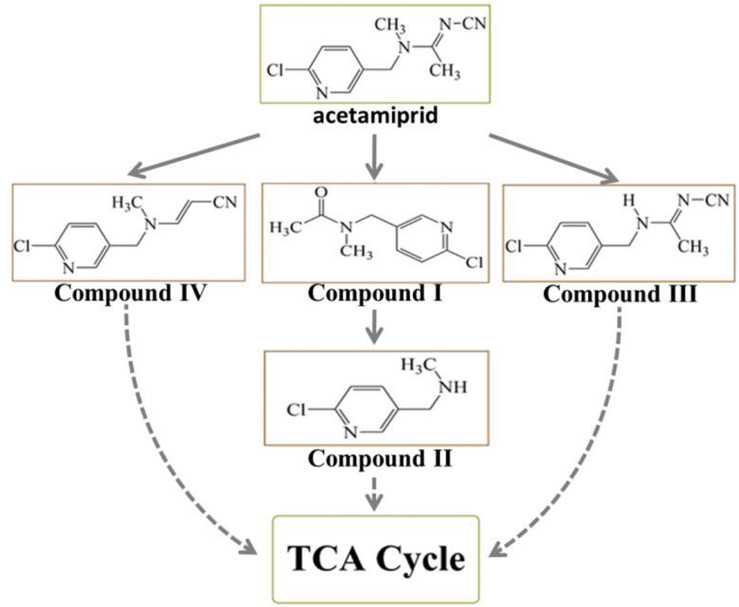
The proposed metabolic pathway of acetamiprid degradation by consortium ACE-3. The dotted line indicates a putative metabolic pathway.

To decipher the metabolic network of acetamiprid biodegradation, HPLC and HPLC-MS were employed to identify metabolic intermediates and propose the pathway of acetamiprid biodegradation by ACE-3. As the profile of the HPLC-MS analysis (Figure 3) indicated, acetamiprid was observed in the control sample. Mass spectrometry revealed the presence of four intermediates in the sample that underwent acetamiprid biodegradation. These correspond to the deprotonated molecules of compounds I {m/z = 163.08 [C5H3N-CH2-N(CH3)-C(O)eCH3]^–^, 154.91 [Cl-C5H3N- CH2-N-CH3]^–^, and 126.00 [Cl-C5H3N-CH2]^–^}, II [m/z = 126 (Cl-C5H3N-CH2)^–^], III [m/z = 194 (M + H-CH3), 167 (M-CH3-CN) and 126 (M-NHCCH3NCN)] and IV [m/z = 194 (M + H-CH3), 167 (M-CH3-CN) and 126 (M-NHCCH3NCN)]. Their respective molecular weights were 194.5, 152.5, 207.5, and 203.5. After comparison with the database, the detected metabolites were identified as N′-[(6-chloropyridin-3-yl) methyl]-N-methyl acetamide (Figure 3A), 1-(6-chloropyridin-3-yl)-N-methyl methaneamine (Figure 3B), (E)-N1-[(6-chloro-3-pyridyl)-methyl]-N2-cyano-acetamidine (Figure 3C) and (E)-3-(((6-chloropyridin-3yl) methyl) (methyl) amino) acrylonitrile (Figure 3D). Notably, compound III was found to be identical to the reported findings for IM 2-1 (Chen et al., 2008). Based on the identified intermediates, a plausible metabolic pathway for the biodegradation of acetamiprid was proposed as illustrated in Figure 4.

Because the intermediate compound I was detected in the culture broth, it stands to reason that consortium ACE-3 is able to oxidatively cleave acetamiprid. The formation of compound I by the oxidative cleavage of acetamiprid is consistent with the findings for the bacterial strain *Pigmentiphaga* sp. D-2 (Yang et al., 2013). In addition, as Dai et al. (2010) previously reported, *Rhodotorula mucilaginosa* IM-2 can also transform acetamiprid into compound I. Subsequently, Compound I could be N-deacetylated to compound II, which was also identified in *Stenotrophomonas* sp. THZ-XP and honeybees (Brunet et al., 2005; Tang et al., 2012). The cyanoimine (= N-CN) moiety of acetamiprid gives it a much higher affinity for the nicotinic acetylcholine receptor (nAChR) of insects over that of vertebrates, leading to paralysis and death of insect pests without harming humans and livestock. Furthermore, the loss of the nitro or nitrile group can completely reverse the selective toxicity of neonicotinoids (Tang et al., 2012). Compounds I and II, which apparently lack the cyano-substituent, might be relatively less harmful to mammals or insects than acetamiprid, even if they are released back into the soil. This is obviously beneficial for the removal and detoxification of the pesticide from soil and plants.

The major metabolite compound III was also reported in earlier studies on acetamiprid degradation. *Stenotrophomonas maltophilia* CGMCC 1.1788 can de-methylate acetamiprid to form IM2-1, but the transformation of either the nitro or the cyano group was not observed (Chen et al., 2008). Brunet et al. (2005) also studied some associated metabolites present in honeybees exposed to the pesticide. In the process of co-metabolic biodegradation of acetamiprid by *Pseudoxanthomonas* sp. AAP-7, Wang et al. (2004) also identified the metabolite B, which was produced by demethylation of acetamiprid, as well as compound IV.

#### Analysis of the Bacterial Community

**FIGURE 5:**
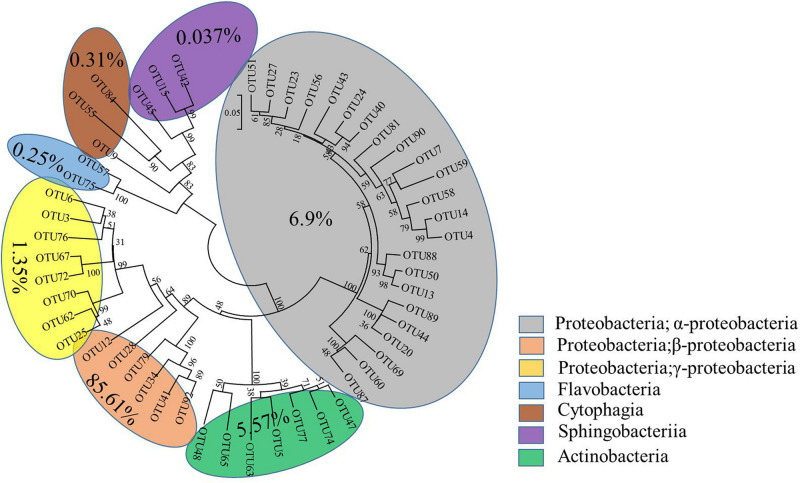
Phylogenetic tree based on the HTS of the acetamiprid-degrading consortium ACE-3. The scale bar indicates 0.05 substitutions per nucleotide position.

The interactive behavior of bacterial populations can affect the degradation of pesticides. Accordingly, further exploration of community diversity and phylogenetic structures of the bacterial consortium ACE-3 through HTS was carried out. After the non-repeat sequences were extracted from the optimized sequence pool, there analysis yielded 40832 valid sequences. The average length of the 16S rRNA gene sequences derived from the community of ACE-3 was found to be 376 bp. To gain an overview of the most likely acetamiprid-degrading populations, the classification and proportions of bacterial species in ACE-3 at the phylum level based on OTU sequence analysis was conducted as shown in Figure 5. In total, seven phyla were identified, whereby α Proteobacteria (6.9%) and β Proteobacteria (85.61%) were the dominant phyla in consortium ACE-3. At the class level, 98.35% of sequences were assigned, with *Actinobacteria*, *Sphingobacteria*, *Cytophagia*, and *Flavobacteria* accounting for 5.57, 0.037, 0.31, and 0.25%, respectively.

Further analysis was carried out to investigate the bacterial community abundance at the genus level, and a total of 51 genera were identified in the bacterial community of consortium ACE-3. To characterize the species diversity of the microbial community, the Shannon’s Diversity Index (H′) (Shannon, 1948) at the genus level was used as a performance index to characterize the diversity of species (OTUs) in consortium ACE-3. Shannon’s Diversity Index was calculated using the formula:

$${\text{H}}_{S\,h\,a\,n\,n\,o\,n}^{\prime }=-\sum_{i=1}^{S_{o\,b\,s}}P_{i}\,L\,n\,P_{i}$$

where S_*obs*_ is the total number of species (OTUs) and P*_*i*_* is the fraction of the total number of individuals in a particular genotype or taxon *i*. The values for H’ range from 0 (low diversity) to 5 (high diversity), and can directly represent the richness of different OTUs in the bacterial community. After calculation, Shannon’s diversity index reached 3.11, which indicated that consortium ACE-3 was rich in diversity.

The dominant genera are summarized in Table 2. These included *Sphingobium*, *Acinetobacter*, *Afipia*, *Stenotrophomonas*, and *Microbacterium*, which respectively accounted for 3.07, 10.01, 24.45, and 49.12% of the total population. Obviously, some of these genera play a key role in pollutant degradation and can be regularly detected in various contaminated environments. For instance, *Sphingobium fuliginis* HC3 was identified as a novel biodegrader that can mineralize biphenyls and polychlorinated biphenyls, while no significant accumulation of derived intermediates was observed (Hu et al., 2015). *Stenotrophomonas* species are widely used for the biodegradation of various aromatic and chlorinated aromatic organic compounds. For example, *Stenotrophomonas maltophilia* D310-3 has the ability of efficiently degrading chlorimuron-ethyl, producing eight degradation products (Zang et al., 2016). *Enterobacter* sp. and *Acinetobacter baumannii* can degrade pesticides and pesticide intermediates, such as imazamox, *p*-hydroxyphenyl acetate, and phenol (Sharma T. et al., 2014; Liu et al., 2016; Thotsaporn et al., 2016).

**TABLE 2:** BLAST results of the dominant OTUs from consortium ACE-3.

OTUs	Percentage (%)	Blast results^a^	Accession nos.	Max ident (%)
OTU28	10.01%	*Acinetobacter baumannii* ATCC 19606^T^	ACQB01000091	100
OTU14	24.45%	*Afipia massiliensis* CIP 107022^T^	AY029562	100
OTU62	0.49%	*Xenophilus aerolatus* 5516S-2^T^	EF660342	98.93
OTU12	0.84%	*Enterobacter cancerogenus* LMG 2693^T^	Z96078	100
OTU47	49.12%	*Microbacterium laevaniformans* DSM 20140^T^	Y17234	100
OTU51	5.42%	*Mesorhizobium cantuariense* ICMP 19515^T^	KC237397	98.14
OTU41	1.03%	*Pseudoxanthomonas indica* P15^T^	jgi.1118276	100
OTU50	1.59%	*Rhizobium pusense* LMG 25623^T^	jgi.1102370	100
OTU60	3.07%	*Sphingobium yanoikuyae* ATCC 51230^T^	JH992904	100
OTU92	0.82%	*Stenotrophomonas maltophilia* MTCC 434^T^	JALV01000036	100
Total	96.84%			

**^*a*^The “^*T*^” after strain names represented type strain.**

In order to elucidate the mechanisms of pesticide biodegradation, the bacterial diversity of many pesticide-degrading consortia has recently been deciphered by different methods. For example, a thiabendazole-degrading consortium was found to contain the *Sphingomonas* phylotype B13 as the major degrading member via denaturing gradient gel electrophoresis (DGGE) analysis (Perruchon et al., 2017a). The propanoyl-degrading consortium T4 was found to contain 59 OTUs using restriction fragment length polymorphism (RFLP) (Hou et al., 2015). Interestingly, these results indicated that the species diversity of bacterial consortia capable of degrading different pesticides, which utilize xenobiotic compounds as the carbon source, became limited after serial enrichment (Zhou et al., 2012).

In recent years, biodegradation technologies have been shown to be viable processes for the complete mineralization of chemical pesticides. For the biodegradation of acetamiprid, increasing numbers of mixed cultures have been screened to study their degradability, including the consortium ACE-3 introduced in this work. However, most of these studies were carried out in the laboratory, which is quite different from the actual degradation in the open environment. Notably, the nutrient conditions in the natural environment are relatively inadequate, and cannot guarantee the efficient growth of bacteria with the desired degradation ability. Moreover, the more complex degradation conditions (e.g., swings of temperature, pH, and oxygen) may be unfavorable to the screened mixed bacteria cultures (Shi et al., 2018). In addition, the nutrient competition and even parasitism by other microorganisms may lead to instability or even the disappearance of the biodegrading bacteria (Ramanan et al., 2015). These disadvantageous conditions are both a bottleneck and a motive force of further research. These problems inspired the exploration and transformation of exciting new technologies, such as microbial modification and evolution, multicellular immobilization and microfluidic screening (Emelyanova et al., 2017; Chen et al., 2019). However, there is no doubt that the microbial consortium ACE-3 screened in this work has great application potential for acetamiprid removal in actual contaminated soil and water environments in the future.

## Conclusion

The natural consortium ACE-3 was gradually acclimatized, enriched and isolated based on its ability to use acetamiprid as the sole carbon and energy source. Possible metabolic pathways for acetamiprid degradation were proposed via intermediate identification. In addition, species diversity analysis was implemented to reveal the changes of community structure and phylogenetic associations. This study provides detailed information on a novel microbial consortium with promising capability for acetamiprid degradation, offering a theoretical basis for further studies on biostimulation or bioaugmentation for *in situ* or *on-site* bioremediation of pesticide-contaminated environments.

## Data Availability Statement

All datasets generated for this study are included in the article.

## Author Contributions

BX, RX, and JZ performed the experiments, analyzed the data, and wrote the manuscript. XW and ZS helped to modify the graphs. FX, WZ, and MJ assisted the manuscript checking. WD provided assistance and guidance throughout the research. All authors contributed to the article and approved the submitted version.

## Conflict of Interest

The authors declare that the research was conducted in the absence of any commercial or financial relationships that could be construed as a potential conflict of interest.
